# Proposed Reforms in the French Metropolitan Asylums

**Published:** 1862-10

**Authors:** H. J. Manning


### Art. II.—PROPOSED REFORMS IN THE FRENCH METROPOLITAN ASYLUMS.

(*Translated from the Original by H. J. Manning.*)

[In December, 1860, a Commission was instituted by the Prefecture of the Department of the Seine, to inquire into the ameliorations and reforms requisite in the provisions for the care of Lunatics in the Department. The members of the Commission were MM. Ferdinand Barrot, Herman, and Amadée Thayer, Senators, members of the departmental Commission of the Seine, and of the Council superintending the Administration of Public Aid; Chaix d'Est-Ange, Procureur-Général attached to the Imperial Court; Véson, Deputy; Marchand, Counsellor of State; Baron Paul Dubois, Dean of the Faculty of Medicine—all four members of the departmental Commission of the Seine; Husson, Director of the Administration of Public Aid; and Dr. Girard de Cailleux, Corresponding Member of the Imperial Academy of Medicine, Inspector-General of Lunatics in the Department of the Seine, (Secretary of the Commission). The following is the highly interesting and important Report prepared by the Commission, and presented to the Prefecture by M. Ferdinand Barrot:]—

Gentlemen,—The law of the 30th June, 1838, imposes upon each department the *obligation of possessing an establishment specially destined for the reception and care of Lunatics, or of arranging to this effect with a public or private establishment, either of the department itself, or of some other department.*

This obligation ought to be accepted by the Department of the Seine in its widest and most liberal sense. We may, however, justly express astonishment that here, where the greatest events of civilization are realized, in the focus of every solicitude, and by the side of those establishments, so numerous and so diverse, in which each of the social miseries finds a refuge, the duty imposed by the law of 1838 has been, if not forgotten, at least narrowly interpreted and carried into effect.

Is it fitting that the Department of the Seine should continue to borrow, for its lunatics, the divided hospitality of establishments having a different destination, and to board them in distant asylums erected by other departments?

In taking under charge, in keeping under its own direction the care of its lunatics, the Department of the Seine would re-enter into its state of habitual activity and its initiative mission. It is natural, then, that it should undertake to found an institution which should include whatever has been accomplished either in scientific knowledge, or the administration of asylums.

It was for the purpose of ascertaining all the elements of this great work that M. the Prefect of the Seine, by a decree of the 27th December, 1860, formed the Commission in the name of which this Report is presented.

The inquiry has embraced every question in the intricate organization of the system which it is necessary to regulate. The Commission have received the aid of those learned alienists who have elevated to so high a pitch this portion of medical science; they have among themselves administrators who best know in what manner to practise the difficult art of effecting benefits; and they have carefully considered what resources would be disposable, and of which it is always requisite to know the extent, if we would not fail of our object by overrating the means to attain it.

They have felt, besides, sustained and fortified by the sentiment which attaches to the work to which they had to apply themselves. Were they not, in fact, face to face with the most poignant of all those miseries which reveal the feebleness of human nature?

Whether the body of man be racked by pain, wasted by infirmities, or bent by old age; whether his soul be broken by despair, exalted by passion, or degraded by ignorance or vice, it is an accident natural to his existence. We deplore it, but it rests within the compass of sense and reason; we comprehend it.

But the soul which is plunged into madness, which has lost its light divine, which has but vague and far-off glimmers; the chain of ideas which is broken, and of which the links are scattered; those remembrances dispersed; those exiled affections of the heart, and even that enfeebled, and at times effaced, conception of God, as if the Creator had turned away from the creature—can we do other than look upon all these things as the most cruel, the deepest, the most grievous of all infirmities? Do they not demand every care, every effort, every sacrifice, and the utmost devotion to their solacement?

Until the termination of the last century the legislation of our country was little occupied with lunatics. It shunned, as it were, these miserables, who inspired rather disgust than commiseration. There were then seen beings deprived of reason wandering upon the public way, subjected to derision, and ill-protected from bad treatment. When their madness, becoming furious, endangered public order, they were cast into prison or into some forsaken corner of a hospice. The law had not foreseen or prescribed anything relating to the sequestration of these unfortunates, the state of which, for the most part, was dealt with arbitrarily and regulated as a question of police.

The first law devoted to lunatics is that of the 24th August, 1790; and this solely entrusts to the municipal authority *the charge of obviating or of amending the troublesome accidents which might be occasioned by the insensate or furious left at large.*

Since the law of 1790, whenever the legislator has occupied himself with lunatics, he has considered the protection of society from the troubles which might be occasioned by them, rather than their protection from others or from themselves, their comfort or their cure. It is only in the Code Napoleon and the Code of Civil Procedure that is met any trace of a just protection for the person or the interests of the lunatic.

The law of 1838 introduced a new principle. It regarded mental alienation, not only as a public danger which it is necessary to remove, but also, and particularly, as an infirmity or a malady which it is needful to relieve; it was the indication that society had taken up this misery, until then neglected, and had given it the right of citizenship in the wide domain of public aid.

"In adopting," said the Marquis de Barthelemy, reporter of the law to the Chamber of Peers, "all the measures which tend to procure for the unhappy lunatics more numerous asylums, and a more rational treatment; in causing to disappear from our Codes prescriptions, the accomplishment of which would have been prejudicial to their cure; and in encompassing their person and their property with every solicitude, the law acquits the debt of humanity."

It is under this idea of good order, justice, and charity that unquestionably the organization of the measures relating to lunatics ought to be investigated.

It is just, first, to state that the present service of the insane of the Department of the Seine is as satisfactory as comports with the insufficient conditions of its organization.

Placed under the direction of the Public Aid of Paris, it participates in the advantages of this Administration, so devoted to its duties, so exact and active, and which, although firmly attached to its old and respectable traditions, nevertheless marches resolutely in the way of progress, applying itself always to protect from all precipitation and temerity the interests which it directs. During sixty years this Administration has successively introduced into the service of the insane the material ameliorations which experience indicated, and it has not depended upon it that these ameliorations were not more promptly and more largely realized.

But that which we owe to it above all things, is the high degree to which alienist science has been brought in the two asylums of Bicêtre and Salpêtrière. There the great school of this science is formed, and those who have or who still profess it there, have always merited, as well in the medical world as in public opinion, the most legitimate renown and incontestable authority.*

Let it be permitted to us to indicate in a few words the progress which has been made up to the present time.

Before the Revolution, the insane of Paris were divided into two categories, the *curable* and *incurable*. Two wards at the Hotel Dieu were set apart for the treatment of the curable, the one for men the other for women. The administrative reports of the time give us to understand that the ward Saint Louis, that of the men, contained ten beds each to contain four persons, and two small beds; the ward Saint Martine, for women, contained six great beds for four each, and six little beds. Thus there were

---

* It suffices to name of these illustrious and honoured men—Pinel, Esquirol, and Ferrus, among the dead; MM. Lelut, Trélat, and Baillarger, among the living.

laid, or rather were bound down upon the same bed four poor insensates, who were irritated by the incessant excitement of this odious community, and whose malady, under such conditions, curable perhaps by calm and isolation, rarely ceded to the treatment followed. Some places in these wards were expressly reserved for hydrophobia.

Mental alienation was associated and placed in contact with canine madness!

In these contracted, infected wards lived, ill-treated by their guardians, this sad population. The furious were not separated from the peaceable, and a nearly uniform treatment was applied to all. The majority remained fastened to their beds for an indefinite period. There was no yard where they might respire better air, not even a covered walk, and consequently exercise was not possible.

As to the insane reputed incurable, they were distributed, according to their sex, either at Bicêtre or at the Salpêtrière. The cells at Bicêtre were formerly destined for criminals; they were only six feet square and received light solely through the doorway; the straw of the bed to which the lunatic was chained was putrefied and rarely changed. The cells of the Salpêtrière were even worse still; there were some placed three metres beneath the surface of the soil, on a level with the sewers, icy and streaming with water. . . . . .*

Forty-four places were also set apart at the Petites-Maisons, now the Hospice Menages, for the reception of incurables able to pay for their board from 400 to 500 francs.

But in all these establishments, the hygienic management and medical treatment were in the most neglected and irrational condition. La Rochefoucauld-Liancourt, in his report to the General Council of Hospitals (1791), paints, with bitter eloquence, the lamentable picture of the shameful state of degradation and culpable neglect to which the indigent lunatics were reduced.

At present, the poorer lunatics of the Department of the Seine are treated in two asylums, which are only annexes of two large hospitals for the aged: Bicêtre for men, La Salpêtrière for women.

Patients for whom there is not room in these two asylums are transferred to 17 asylums in different districts not in the Department of the Seine, and sometimes at considerable distance.

On the 1st January, 1861, the population of these different establishments amounted to 4213: of whom 1700 were men, and 2513 women. They were distributed thus:

---

* These details are taken from the *Report of Pastoret to the General Council of Hospitals* (1791), p. 117; and from the *Report of Camus* (Fructidor, an X.) p. 82.

| *Male.* | | *Female.* | |
|---|---|---|---|
| Bicêtre . . . . . . . . | 903 | La Salpêtrière . . . . . | 1362 |
| Other Asylums . . . . . | 797 | Other Asylums . . . . | 1151 |
| Total . . . . . . . | 1700 | Total . . . . . . . | 2513 |
| Grand Total . . . . . . | | 4213 | |

It was not till 1807 that the service of the insane at Bicêtre and at the Salpêtrière was organized; and notwithstanding the efforts of the General Council of Hospitals, and the repeated protests of the Medical profession, it remained a long time subject to the unhealthy and inefficient conditions which the official reports disclose.

In 1826, to the great benefit of humanity, the cells which we have described were replaced by chambers. There is, in the two words "*cells—chambers*" an indication of the new spirit which was about to pervade the treatment of lunatics. The principle of treatment was being modified. In place of those barbarous means of coercion which were employed in most cases, and which showed the absolute degradation of the patient and the inability of the surgeon, a system of gentleness, of intelligent and compassionate superintendence, was being introduced, which, if it did not cure the afflicted, guaranteed them at any rate a happier existence, and left them perhaps some vague feeling of their dignity as human beings.

Pinel had already commenced this salutary revolution in the mode of treatment of lunatics. After him, his worthy successors Esquirol and Ferrus (I speak only of those who are gone), have followed him in their practice, and at the present day the professors of the science are applying themselves zealously to the development and perfecting of this system.

The lunatic service of Bicêtre is divided into three sections, each being under the superintendence of a head physician.

The two first sections are each subdivided into two: one appropriated to the violent and occasionally violent patients, the other to the quiet and dirty, and containing also an infirmary for incidental attacks of illness. The third section contains epileptic and idiot patients, both adults and children, with the interior divisions necessary for so diverse a population.

At the visit of the Commission to Bicêtre, under the presidency of the Prefect of the Seine, he was at once convinced that, in spite of the care employed in the maintenance of the buildings appropriated to the service of lunatics, the material conditions of this institution were opposed to a proper hygienic system, and to the rational treatment of mental disorders. Air, light, and space were wanting in these buildings, which belonged to several epochs, and were originally constructed for very different purposes. On the ground floor, the rooms were nearly all dark and damp, the parlours narrow and uncomfortable: on the higher floors were dormitories with low ceilings, encumbered with beds, badly aired, and sometimes placed just under the roof, and consequently cold in winter and scorching in summer;—the doors low, such as were necessary for the former inmates of these building;—four-story buildings, with narrow twisting staircases, where persons with all their faculties about them find some difficulty in avoiding the too narrow landings, the too sudden turns, and the high projections. It is an exception to meet a room well lit and of proper size, or a garden with a little green in it and a country view. Indeed, this whole pile of old prisons and hospitals with some modern buildings added, which it has been found difficult to apply properly to their use, has a general appearance of unfitness and discomfort. It seems impossible that poor disturbed intellects should find in such a place the quiet and calm which springs from a well-ordered state of things.

Besides the three sections of which we have just spoken, there is a division attached to one of the extremities of the asylum, intended for accused or condemned persons who have been remitted by justice for medical examination, as afflicted or threatened with mental disorder. In this building, the faulty arrangement of which is justly criticised, are confined also persons whom medical men have pronounced to be dangerous.

At a distance of about a mile and a quarter from Bicêtre, Dr. Ferrus has instituted an asylum known as St. Anne's Farm. M. Ferrus had organized there certain works of cultivation and of agricultural industry, in which at first only convalescents from the three sections of Bicêtre were employed, but which now employ a certain number of chronic patients. The cultivation of the farm properly so called, was without real importance. Some patients were hired for agricultural work on neighbouring farms: but the principal occupation at present is the management of a piggery, the products of which have been for some time the clearest profit arising from the farm.

The population of female lunatics comprises at the Salpêtrière five sections, which are subdivided, as at Bicêtre, into wards in which the different categories of patients are treated. Here, as at Bicêtre, the Commissioners were struck with the inefficiency and faulty arrangement of the building. The inconveniences of all kinds noticed above are met here on every side, and frequently in a very reprehensible degree.

At each of the two asylums there are workshops, in which the eligible patients, male and female, are employed in different industrial occupations. It will be seen that this work being dependent upon a state of tranquillity, and proportionate to the strength of the patient, is of a very irregular character, difficult to manage, and of very variable profit.

About a third of the population of Bicêtre is thus employed: 113 patients, children and adults, labour at cultivation and various agricultural works; 245 at trades, or house work, such as washing, cleaning, cooking, &c. They receive pay in proportion to their work. In 1859 the remuneration amounted on the whole, for the year, to 15,587 francs, being an average of 43f. 52c. a-head.

At the Salpêtrière, 993 patients, more than three parts of the population, were employed, in the year 1859, in the workshops and in house work: their pay amounted to a total of 40,942 francs; an average of 41f. 23c. a-head.

In the 17 asylums which are not in the department, 997 males and 1151 females were under treatment at the beginning of this year (1861). These are patients, as we have seen, for whom room could not be made at Bicêtre or La Salpêtrière. The transfer of this excess, which represents almost the half of the whole number of patients of the Seine, is determined by very vague rules with respect to the choice of patients to be transferred.

Of course the intention is to transfer only those whose chronic state appears to admit of little or no chance of recovery; or those who have no friends, or have been deserted by their friends. But it is impossible to believe that in such a large number of patients all can be included under these heads. All certainly are not incurable, for statistics show that some are returned cured. All are not friendless, for some are visited by their relations or have been in correspondence with them at first, although on account of the distance their correspondence has gradually diminished in frequency, and at last ceased entirely. It is a painful reflection that there are consequently among these unhappy persons some whose family ties have been violently torn asunder, and in whom the affections, which are the first and last manifestations of social life, have been allowed to die out. They may have felt, in some awakening natural feeling, that they were being torn from familiar scenes, and from the tender compassion of those who love them still. And perhaps the pain and despair of such an exile may have flashed across their troubled intellect.

It is a necessity, however, that must be submitted to. So long as the Department of the Seine does not build special asylums in numbers sufficient to provide for all its patients, distant hospitality must be hired to take charge of them.

It would take too much space, and be perhaps difficult to give an exact account of the organization of the asylums of which we speak, and of the treatment adopted there. They are for the most part public asylums founded by departments in accordance with the law of 1838; the rest are private asylums. Many of them have acquired a just celebrity for their excellent arrangements, and the activity and intelligence with which they are directed.

One of them, which was visited by the Commission, has been the object of close study. It was founded at Auxerre by the authorities of the department, and with the assistance of one of our learned colleagues, M. Girard de Cailleux. When Baron Hausmann was Prefect of the Yonne, he prosecuted the building and reorganization, and prepared the asylum for its future career with that prompt decision and vigorous initiative which we have since seen employed on the grandest subjects.

The committee, on its visit, was so favourably impressed by the asylum at Auxerre, that they thought fit to propose it as the type which it would be most desirable to imitate in building the asylums of the Department of the Seine.

Without inquiring particularly into the administration or medical treatment adopted in the asylums to which our surplus patients are transferred, but confining ourselves to the general remarks already made, we say that this remote hospitality is not fitting for a great and rich department like ours, either in respect of the liberal execution of the law of 1838, or with regard to the proper relief of our patients, or the fulfilment of our duty towards the unhappy beings placed in our care.

The Commission had to do only with asylums for the indigent. With regard to lunatics whose families are able to pay for their treatment, numerous asylums are open to them, and the State has provided the special institution of Charenton, where patients are taken in at regular charges.

The above is a general description of the actual organization of the lunatic asylums of the Seine. Owing to the defects, the inefficiency, and the grave imperfections revealed by this organization, the Prefect of the Seine, in a memorial addressed to the General Council at its last session, summed up the state of things thus:—

"The Department of the Seine possesses no special asylum for lunatics. A great number of lunatics are very intelligently and attentively taken care of in two hospitals, which are chiefly devoted to other uses, Bicêtre and La Salpêtrière; but their position there is necessarily incomplete, defective, and not in keeping with the requirements and progress of medical science. The classification of patients according to the nature of their malady, cannot be carried out in the manner necessary to facilitate their recovery. Besides this, the insufficiency of the accommodation compels the department to find room for more than a third of its patients* (1661 out of 4030), in a score of asylums scattered over the whole of France. The moral and physical treatment is excellent in most of these asylums; in other aspects, the rules of admission, of food and clothing, and of medical attendance, are more or less imperfect. Besides, it is always an evil to separate a lunatic far from his family, especially a pauper lunatic. They do not visit him, and he is completely abandoned, and the feeling of desertion may make him incurable; consequently, the patients of the Seine are less easily cured in the extra asylums than at Bicêtre and La Salpêtrière, deficient as these hospitals are in some respects. Such a state of things cannot be allowed to continue, and next year, gentlemen, I shall propose to remedy them." This promise, which was hailed cordially by the Council, it is our business now to fulfil.

The end proposed by the resolution which instituted the Commission was this: To secure for the treatment of lunatics in the Department of the Seine satisfactory conditions and a direct and special administration. With this object, to build special asylums, comprising all the interior divisions necessary for the separate categories between which it is useful to make a distinction. Recognising the fact that the asylum by its material arrangements is one of the most powerful agencies in the cure of madness, to endeavour to combine for the comfort, well being, and cure of the patients, a good style of building with easy and lofty passages, a pleasant and quiet situation, and an open sky; to divert these troubled minds which are often besieged by one single idea, by means of regular work, especially work in the fields; to employ them in the business of real life, which brings back to them at intervals will, observation, appreciation of order, and gives them a kind of relative discernment, which occasionally restores insensibly reason to its throne: to assist the progress of alienist science by giving it a more regular and surer abode: to advance the practical teaching of it by means of clinical instruction: to institute thereby a regular school for this branch of the healing art where its illustrious professors might instruct pupils worthy to succeed them: to establish in these asylums the treatment commenced by Pinel, Esquirol, and Ferrus: substituting gentle and compassionate care, which soothes and conciliates, for violence, which irritates and drives to despair; and lastly, to bring the lunatic under the natural and salutary influence of his family, so that his friends shall not forget their duty or desert their affections.

This is the programme which the eminent magistrate, the

* The numbers given in a former page increase the proportion given by the Prefect considerably; they are taken from more recent calculations.

President of the Commission, had laid down for it from the commencement of its deliberations.

In order that it might prosecute its inquiries with confidence, it was necessary to inquire with respect to the resources available for the vast work which it hoped to realize. Assuredly this work is in itself of such great interest and so urgently required, that the expense of carrying it out might justly figure among the most legitimate expenses of the budget of the Department.

But we may state at once that this cost may be provided for. The Prefect has informed the Commission that a sum of ten million francs, from the surplus funds of the Bakehouse Bank, can, subject to certain legal powers being obtained, be set apart for the expenditure which would be rendered necessary by the systematic organization of the requisite provision for the insane.

We are fortunate in being able to prove, on this occasion, the prosperity of this institution (owing to the firm initiative taken by the Emperor), which has in spite of all difficulties worked so well, that it has now sufficient credit and money to sink ten millions in the budget of public charity.

The first question was to know the real number of the lunatic population for whom the Department of the Seine had to provide.

We have said above that on the 1st of January, 1861, the number of lunatics in the department was 4213; but it would not be prudent to take this number as the base of a definite organization. We may, indeed, expect a more and more rapid increase in the number of lunatics. If this increase is already well marked in the whole of France, it is still more so in the Department of the Seine. At the commencement of the year 1801, the number of lunatics in the hospitals and asylums of Paris was about 946. In fifty years it was more than tripled, and reached 3061. During the last ten years it has increased by more than a third (4213). From 1800 to 1851, the increase was at the rate of 42 every year: from 1851 to 1861 the increase was 100 a-year.

Whether we attribute this progression in a great measure to the increase of general population, or to the plan of transferring lunatics, which lessens sensibly, and for different reasons, both the cure of patients and their being taken away by their friends uncured; or whether we look for an explanation higher, in political agitations, in the change of habits brought about by the feverish agitation of public affairs, which wearies and exhausts the mind, is of little consequence; the fact must be taken into account, and the commission, allowing for future circumstances, has fixed 6000 as the number on which to base their calculations. It would be impossible to think of shutting up so vast a population in one establishment. The administrative and medical requirements would not admit of it.

The rational treatment of lunacy is based on the special, close, and almost personal attention required by each patient, especially in acute and recent cases. All the varieties, the progress, relapses, and changes of the state of mind, should be followed step by step. The doctor should read as it were constantly the vacillating reason which flashes and sinks by turns. This appears an almost ideal plan of treatment; but of course, while leaving impossibilities out of the question, we must endeavour to attain in practice what will best ensure an efficacious line of treatment. On this account it is that in all well ordered asylums the categories are numerous, carefully separated, and enclosed. In an over-filled establishment, each of these categories would become too crowded for the proper carrying out of the treatment, and the doctor could not do his duty thoroughly.

What, then, would be the best and most useful number of establishments among which to divide the whole body of patients? After a long and searching discussion, the Commission, anxious to provide for the requirements of science and the exigencies of finance, resolved upon a system which gained universal consent, and which we will explain.

It is admitted that the maximum of patients to be admitted into one asylum should be 600. This number allows a suitable division into categories, complete administrative surveillance, an easy keeping of accounts, and a sufficiently watchful medical attendance.

Of course, in an economical point of view, it would be desirable that asylums should be more crowded and less numerous. It appears indeed that, for the purchase of land, the building, and internal arrangements, it would be less expensive to build a single asylum for 1000 or 1200 patients, than two asylums for 600 each. But what is true up to a certain point for houses of refuge and hospitals, is much less true for a lunatic asylum. In this latter case, we repeat, the interior divisions are required by the exigencies of medical treatment. Each division is, so to speak, an establishment apart from the rest, with its own special and exclusive conditions; the number of its inmates should be necessarily limited, for in almost every category a large number would constitute danger. Can we conceive, for instance, the furious or semi-furious being together in large numbers? On the other hand, in one building there is something gained in the greater extent of each of the interior divisions. Unite several of the squares of a draughtboard to make one large square, and you economize all the old divisions; but if you are obliged to preserve the size of each square, you gain much less by making the draughtboard larger.

With regard to land, certain fixed space is necessary for each asylum, under certain conditions which will admit of the institution of open-air work in its most useful form, as we shall see presently. Setting aside the fact that it is much easier to procure a moderate extent of land near Paris, it must be remembered, that in order to be efficacious this agriculture must not be carried on in too large a space, large numbers and extended fields being both inconvenient and dangerous.

Again, there is one item in the general expenses connected with lunatic asylums which increases in exact proportion to the number of the inmates. Every keeper has to some extent a medical duty: he must be attentive, watchful, active, kind, but firm, in his dealings with these grown-up children, who are always restless, troublesome, and complaining; falling now into a faint, now into a sudden fury. Of course the exercise of such intelligent and unceasingly watchful care must be necessarily limited. In a word, all the exceptional conditions connected with an establishment of so exceptional a nature must be taken into account, and hence it is clear that measures of economy which are useful and practicable in other undertakings are less so in this.

The distribution of the patients in these asylums comprises a whole system of infinite delicacy carefully laid down by science.

In the old establishments we have seen that a brutal and frequently blind division was sanctioned into curables and incurables: the former remaining under the eye of science objects of its study and care; the latter forming merely a herd, left to the chances of animal life, regarded by science, if not without pity, at any rate without hope or effort.

Doubtless, in examining the statistics, one is shocked at the small per-centage of cures in mental afflictions. But modern science and humanity must not hesitate before these cruel figures. For them every suffering may be soothed, or is worthy the attempt. They search undismayed for a last spark in the ashes of the intellect that is given up for dead. They never despair; but armed against the malady, they watch and strive, like the mother who, to the last, gazing on her child, hopes for a miracle to repay her care and love. But if this division into curable and incurable is no longer allowed as fundamental, we must yet admit that the distinction is too clear to be entirely passed over.

Studying at the same time the comfort of the patients and the progress of science, the Commission recommends that a Central Asylum should be founded at Paris. Placed thus in the midst of the intellect of the capital, officered by the most eminent professors of the science, and presenting for study every variety of mental disease, this asylum would become the very home of alienist science. Here new methods would be received with caution for the test of experience; here would be centred the great practical teaching of the art of curing or alleviating mental disease. As yet this instruction has not been of a practical character, and the object is to make it so by establishing a true clinical course. To this introduction of a clinical course of instruction into lunatic asylums, some objections will be naturally raised. The large concourse of students, their studious, but sometimes ardent curiosity, the imprudent manifestations of their impressions, and other unavoidable concomitants of clinical instruction—are these compatible with the proper treatment of lunacy? Would the patient bear without harm the presence of a number of assistants, explanations of his disease (which he himself always denies the existence of), and other practical demonstrations? All these things which are readily submitted to in our hospitals by the greater number of patients, who perceive that by assisting the researches of the science which heals them, they are contributing their share toward the relief of their fellow-beings; would not all these things, we say, be misunderstood and refused by these persons whose reason is distrustful in proportion to its weakness?

Of course it would be indispensably necessary to introduce in the clinical instruction at the central asylum certain special proceedings. Clinical instruction has already been attempted by several professors, as Esquirol, Ferrus, Leuret, Falret, and Baillarger; and by means of great reserve and caution they were successful. Certain rules then should be laid down, but it must be left principally to the tact of the learned professors to determine their line of proceeding.

We have said that in the Central Asylum every variety of mental disease would be represented, recent and acute cases as well as chronic, whose successive developments, lesions, and changes it would be useful to study. It will be divided into four departments: each department will contain its different categories, and will be under the care of a physician, as is the practice at Bicêtre and La Salpêtrière. The superintendence will be entrusted to a general manager not chosen from the medical profession.

The Commission insisted specially upon ensuring from the commencement a character of unity for the different departments. To attain this end they recommended that there should be an office of admission as an annexe to the Central Asylum; where those who possessed the regular qualifications, or who were brought, either owing to an order from the Prefect, or the wish of their friends, should be examined. The state of the applicant being determined, he should then be, according to circumstances, either kept in the Central Asylum, or sent to one of the other asylums, of which we shall presently speak. At present, lunatics from the public streets, or taken at the instance of their families, or neighbours, are removed to the police office, where they are confined and deprived of the special care which their malady requires from its very commencement. The physicians examined by the Commission all agree in asserting that this cruel state of things sometimes exercises a fatal influence on the subsequent course of the disease. They hailed with marked satisfaction the project of a place for provisional admission as above described, pending the completion of the legal formalities. The dignity of the family and of the individual would be more respected by a prudent and discreet hospitality than by this equivocal and unpleasant detention within the walls of a prison.

These being the principal regulations of the institution in the centre of Paris, the Commission thinks that the other asylums might be placed out of Paris, or some of them even out of the Department of the Seine, if confined within reasonable distances.

The institution of a central and of additional asylums has given rise to several questions which are worth noticing.

Should men and women be received into the same asylum, an absolute separation being kept between the sexes? Yes; for it is clear that this secures in different ways incontestable advantages. Science may draw useful results from the study of the same disease in different sexes. It is therefore useful to compare frequently the results of researches and examinations, and for this purpose the proximity of the two sections is indispensable. An absolute separation between the sexes in the same asylum is easily procurable by the simplest precautions. Many country asylums have adopted this plan, and long experience has shown that none of the mishaps that might have been feared have resulted.

In an economical point of view especially, the question merits investigation. In hospitals, every means of lessening expenditure ought to be sought, in order that while confining the expenses within the limits imposed by the funds, relief may be afforded to the greatest possible number of patients.

The most desirable solution would be that the inmates of an asylum should almost entirely suffice to attend to their own wants. If one sex only is admitted, the stronger among them may be employed in certain occupations and in such profitable employment as may be fitted to their sex. But for other employments and other work, recourse must be had to external aid, which is troublesome and inconvenient, in order to preserve order and discipline in the building. If, on the contrary, the sexes are united in one asylum, they can easily supply nearly all the labour necessary to their common existence. If the number of inmates is kept up to a certain rate, persons of all kinds of trades will be sure to be found among them, and the work done by them will be very considerable. Not only will it lessen the expenditure, but it will add an important item to the receipts.

We have seen above that the Central Asylum will contain chiefly acute and recent cases: but it is natural to wish that a certain number of these cases should be treated in the additional asylums. The system there followed, the open-air life, agriculture, the quietude of the country, are all hygienic conditions tending to hasten the cure of the hopeful cases, as well as to alleviate the sufferings of the chronic. By this mixture of two categories, which were formerly kept so distinct, all traces of the cruel separation of curable and incurable will be effaced, and neither patients, their families, nor the public need read over the entrance to these asylums: "Here, leave all hope behind." And on the other hand, the physician will not be condemned to the thankless and painful task of exhausting the efforts of his science and his care upon patients who have been branded with the fatal mark of incurability.

While each asylum is to be filled with patients afflicted by every form of mental malady, there are yet two of these forms which seem to call for special attention: viz., epilepsy and idiocy. They require special modes of treatment, and carefully studied internal arrangements. All medical men agree upon this point, that it is eminently useful to establish asylums where these two categories should be exclusively received. The Commission has adopted this opinion. In these asylums, the divisions would be regulated by sex and age. Children, placed in a distant part of the asylum, would receive an education corresponding to the nature of the fearful disease which degrades or kills them.

The excellent effect of gymnastic exercises should be specially considered. The Commissioners have seen at Bicêtre children, who having entered the asylum in the state of physical and mental prostration peculiar to idiocy, have by degrees attained a kind of energy both of will and of motion. It appeared that under the influence of these exercises properly directed, the body returned by degrees into communication with the intellect.

Besides the general arrangements to be observed in the formation of new asylums, the Commission turned its attention to the plans of treatment which might be adopted in them. It would be rash to attempt to decide between the different modes of treatment which have been proposed by the learned physicians who have written upon the subject; they belong to the field of science, but we may be permitted to express our confidence in the new modes introduced into several asylums, the excellent results of which the Commission has had an opportunity of testing.

One of the most rational means of cure is assuredly by the open-air life, and the employment of the patient in work proportioned to his skill and strength. One may easily mark the salutary effect produced upon excessively impressionable patients by plenty of air, by the sight of plains, fields, and trees, and all the works of Nature which remain at the same time in unalterable order and in infinite variety, and which, while they attract the mind, calm and absorb it. The personal preoccupation, the feverish and exclusive egotism of the lunatic yield and disappear under the attractive aspect of the country and the distant horizon. Now put instruments into this poor creature's hand: set him to dig and to sow, and then to cultivate the plants which grow and ripen under his eyes, and may we not hope that in the course of this growth the absent intellect may insensibly return, may gain strength by successive efforts, and perhaps attain activity and persistence, just as friction gradually gives strength and flexibility to muscles stiffened by disease.

The Commission had an opportunity of studying the effect of agricultural labour on lunatics. They found the practice in full operation at the asylum of Auxerre and of Fitz James at Clermont (Oise). It was pleasant to see men, who in old times would have been left to their own resources and with no companionship, in a prison, whose pitiless walls suggested incessantly to them one persistent idea, as an additional fuel to their madness, spread over the fields under a cheerful sky and taking the deepest interest in the cattle committed to their charge, or the plants they reared, and in other things and beings who stood in need of their attention. To see them thus employed, they appear to become men again, to rise above their miserable state by means of this protection required from their darkened intellect. If cure does not always result, at any rate there is always an alleviation of suffering and a kind of respite from incurable madness.

The additional asylums, then, must be organized with this particular object, and that part of the regulations which relates to the proper organization of the agricultural and industrial work allotted to the patients will be by no means the least difficult. As the proper use of labour is salutary, so the abuse would be cruel and disastrous. Above all, it is necessary to guard against too freely speculating on the advantages to be derived from the work for the funds of the asylum.

From what has been said, it will be understood what interest attaches to the choice of sites for the asylums. Only at a certain distance from Paris, where the price of land is not exorbitantly high, can be met suitable conditions of site, space, and healthy atmosphere. Everything connected with these questions should naturally be left to the authority of the department. The Commission has merely expressed its hope that they will take into special consideration the facility of communication between the asylums and the capital, in order that the communication between the patient and his family may be as frequent as possible.

Another interesting question has attracted the attention of the Commission. Should the asylums intended for pauper patients be arranged so as to receive, subject to certain conditions, other patients as boarders whose quarters, though completely separated, should yet be really part of the asylum ?

The Commission, for several reasons, thinks in the affirmative.

In a scientific point of view, the medical men examined by the Commission seem to admit that medical observation of similar diseases contracted under different circumstances, and modified by distinct habits of social position, would be of practical advantage to the study. This is probable. But the Commission was influenced by a more positive consideration. Families in the middle class of society cannot always afford the sum required in non-pauper asylums. It is reasonable to suppose that for such families it would be an immense advantage to be able, by means of a scale of charges, to place their patient in establishments organized upon the best principles, superintended by the masters of the science, and under the control of an administration which avoids all the dangers or inconveniences to be apprehended in establishments more or less subject to the chances of private speculation. The separation of these annexes should be so marked as to preclude any idea of communication or confusion in the mind of the public. We can imagine the dislike of families to any association which tells too clearly the nature of the place. But they do not avoid this evil in special houses whose speciality cannot be hid, and whose name is a mark which cannot be mistaken.

The sum paid for board would of course be one of the resources of the chief establishments. What in other cases is private gain would thus be an assistance to public help and humanity in which its relations would be satisfied. This kind of combination has been a complete success in many country asylums, markedly so in the Auxerre asylum, which we quote as an example so frequently.

What should be the principle of superintendence in the new asylums ? Shall they be managed like the present public establishments, where a governor has charge of the administration, properly so called, of the surveillance of the medical staff, and of the arrangement and control of the expenditure? In such a case, the authority of the governor is very great. He has no right, it is true, to interfere in matters which concern specially the medical authorities, but he has a right, indeed it is his duty, to see that the medical duties are carried on in conformity with the rules. Such a state of things involves contradictions and disputes: the requirements of the medical authority may be often opposed to the views of the administrative authority, and when there is not, as at Paris, an arbiter whose authority has incontestable weight, the result is a thorough state of anarchy, prejudicial to the interests of the hospital.

It is clearly desirable above all things that the authority should be single; that the whole, which is called hospital or house of refuge, should be in absolute harmony, and that all the elements, administrative or medical, should concur with one impulse for the common good. This state of harmony is not always obtained, even when under the control of one directing head. A faculty of concentration, of exactness, of correctness, a firm will and an active disposition, are essential qualities which are rarely met united in one person in a sufficient degree for the administration of the most ordinary business.

But if the conduct of an asylum is a complex affair, if it requires the working of two distinct orders of ideas, if it involves the watching over interests sometimes opposed to one another, is there not ground to fear that the intelligence and activity of a single individual cannot be always equal to the task? If it were proposed to invest in the physician both the administrative and medical authority, is it quite clear that he will be equally fitted for both duties? Will he be at once the studious, attentive physician, devoted to his profession, and the superintendent master of all the details, active, firmly sustaining the internal discipline of the establishment, economizing and inventive?

When such a rare man is met with, it is a notable piece of good fortune. He has been already found occasionally, and a few of the country asylums prosper well under the sole superintendence of the physician. The Auxerre asylum was directed for many years by Dr. Girard de Cailleux, who brought it to a state of perfection and prosperity which still continues under his successors. In an ordinary hospital, where there is great variety of disease, and where nothing more is requisite than careful attention to the prescriptions, the division of the ruling power is a rational proceeding. But it is much less so in a lunatic asylum, where the formulæ are more various, where the prescriptions leave a certain margin, and where in some cases the treatment, involving every act of the patient's life, and his liberty, exercise, &c., has to be measured out to him according to the circumstances of the moment. Here it is clear that all the means employed must conjoin to the same end. This question has received, as it deserves, considerable attention at the hands of the Commission. It does not, however, admit of absolute solution. When the authorities of the department think that they can entrust the medical and administrative authority to the same person, as they have power to do by the law of 1838, they will be right in doing so, for such a management will be the nearest approach to perfection.

During the discussion a very good and probably practical idea was expressed, and received serious consideration. There are some quiet, inoffensive lunatics who may be left with their friends; their treatment is very simple, and generally purely hygienic; and the kindness of the patient's relations will ensure the carrying out of the physician's directions. Why not apply to such the system of treatment in their own houses which is applied by the managers of hospitals to other sufferers? This method of administering relief while leaving to the friends their share of duty is receiving every year more attention. It is a good idea, since it tends to substitute the domestic hearth for the hospital, and so avoids breaking the connexion between the patient and his relations, and destroying his domestic affections. It has also the advantage of economy, by limiting to some extent the expenses incurred in the foundation of hospitals. The effect, too, of lessening the expense in carrying out the improvements in the Department of the Seine would be produced, if treatment at their own homes could be carried out with a certain number of lunatics. And why not? The fact is, that there is a whole category of lunatics who, if they are not able to help themselves, might at least be employed to some extent in different household work, or in profitable labour.

How is this new system, founded upon new data, to be realized? It would be a poor acknowledgment of the high intelligence of the government of the Department of the Seine to suppose that the extensive reorganization here projected is to be carried out at one sweep. The work will be a work of time: it comprises measures requiring the exercise of the greatest discretion.

The central asylum must be instituted first; from it will spring gradually the whole reform. The additional asylums will follow in their turn as they are required, and as means will allow. The number of establishments that we have recommended is for a population a third more numerous than at present exists. The Administration has plenty of time before it need proceed to the completion of the entire number which will be required. Everything will be done in a proportion and order which will allow us to verify, so to speak, during the execution of the work, all the progress that has been made and reforms suggested by the practical working of the details.

The Commission were unanimously of opinion that the realization of the projects, the principal elements of which have been enumerated, would best meet both the duty and interest of the Department of the Seine.

The resolutions of the Commission may be summed up thus :—

1. The institution of special asylums for the lunatics of the Department of the Seine.

2. Direct management of these asylums by the Government of the Department.

3. A central asylum in Paris, into which should be admitted all forms of mental disease, but more especially acute or recent attacks, and in which clinical instruction should be carried on.

4. An office in connexion with the central asylum for the reception, examination, and classification of supposed lunatics.

5. Additional asylums, not in Paris, but at distances which shall admit of easy communication between the patients and their friends.

6. Asylums for epileptic lunatics and idiots exclusively.

7. The building of new asylums on a principle which will allow the admission of patients of both sexes, with strict separation from one another.

8. The erection of buildings connected with the asylums, but distinct from them, wherein boarders can be received at fixed rates.

9. The administrative and medical authority to be united in the same individual, if it be possible.

10. Employment of the patients in different kinds of labour, especially in the open air.

11. The adoption of the system of treatment at the patient's own home in all cases where there is no risk of public annoyance.

